# Global Discriminative Learning for Higher-Accuracy Computational Gene Prediction

**DOI:** 10.1371/journal.pcbi.0030054

**Published:** 2007-03-16

**Authors:** Axel Bernal, Koby Crammer, Artemis Hatzigeorgiou, Fernando Pereira

**Affiliations:** 1 Department of Computer and Information Science, University of Pennsylvania, Philadelphia, Pennsylvania, United States of America; 2 Department of Genetics, University of Pennsylvania, Philadelphia, Pennsylvania, United States of America; University of California Santa Cruz, United States of America

## Abstract

Most ab initio gene predictors use a probabilistic sequence model, typically a hidden Markov model, to combine separately trained models of genomic signals and content. By combining separate models of relevant genomic features, such gene predictors can exploit small training sets and incomplete annotations, and can be trained fairly efficiently. However, that type of piecewise training does not optimize prediction accuracy and has difficulty in accounting for statistical dependencies among different parts of the gene model. With genomic information being created at an ever-increasing rate, it is worth investigating alternative approaches in which many different types of genomic evidence, with complex statistical dependencies, can be integrated by discriminative learning to maximize annotation accuracy. Among discriminative learning methods, large-margin classifiers have become prominent because of the success of support vector machines (SVM) in many classification tasks. We describe CRAIG, a new program for ab initio gene prediction based on a conditional random field model with semi-Markov structure that is trained with an online large-margin algorithm related to multiclass SVMs. Our experiments on benchmark vertebrate datasets and on regions from the ENCODE project show significant improvements in prediction accuracy over published gene predictors that use intrinsic features only, particularly at the gene level and on genes with long introns.

## Author Summary

We describe a new approach to statistical learning for sequence data that is broadly applicable to computational biology problems and that has experimentally demonstrated advantages over current hidden Markov model (HMM)-based methods for sequence analysis. The methods we describe in this paper, implemented in the CRAIG program, allow researchers to modularly specify and train sequence analysis models that combine a wide range of weakly informative features into globally optimal predictions. Our results for the gene prediction problem show significant improvements over existing ab initio gene predictors on a variety of tests, including the specially challenging ENCODE regions. Such improved predictions, particularly on initial and single exons, could benefit researchers who are seeking more accurate means of recognizing such important features as signal peptides and regulatory regions. More generally, we believe that our method, by combining the structure-describing capabilities of HMMs with the accuracy of margin-based classification methods, provides a general tool for statistical learning in biological sequences that will replace HMMs in any sequence modeling task for which there is annotated training data.

## Introduction

Prediction of protein-coding genes in eukaryotes involves correctly identifying splice sites and translation initiation and stop signals in DNA sequences. There are two main gene prediction methods. Ab initio methods rely exclusively on intrinsic structural features of genes, such as frequent motifs in splice sites and content statistics in coding regions. Notable ab initio predictors include GenScan [1], Augustus [2], TigrScan/Genezilla [3], HMMGene [4], GRAPE [5], MZEF [6], and Genie [7]. Homology-based methods exploit extrinsic features derived by comparative analysis. For instance, ProCrustes [8], GeneWise, and GenomeWise [9] exploit protein or cDNA alignments, while TwinScan [10], DoubleScan [11], and NScan [12] rely on genomic DNA from related informant organisms. Extrinsic features improve the accuracy of predictions for genes with close homologs in related organisms. Krogh [13] and Mathe et al. [14] review current gene prediction methods.

GenScan was the first gene predictor to achieve about 80% exon sensitivity and specificity in several single-gene benchmark test sets. More recent predictors have improved on GenScan's results by focusing on specific aspects of gene prediction. For example, GenScan++ improves specificity for internal exons and Augustus improves prediction accuracy on very long DNA sequences. Despite these advances, overall accuracy on chromosomal DNA, particularly in regions with low gene density (low GC content), is not yet satisfactory [15]. Gene-level accuracy, which is especially important for applications, is a major challenge.

Improvements at the gene level could have a positive impact on detecting gene-related biological features such as signal peptide regions, promoters, and even 3′ UTR microRNA targets. Genes with very long introns and intergenic regions represent more than 95% of the total number of genes in most vertebrate genomes, and even a small improvement on those could be significant in practice.

With the exception of MZEF, which uses a quadratic discriminant function to identify internal coding exons, all of the ab initio predictors mentioned above use hidden Markov models (HMMs) to combine sequence content and signal classifiers into a consistent gene structure. HMM parameters are relatively easy to interpret and to learn. Content and signal classifiers can be built effectively using a variety of machine learning and statistical sequence modeling methods. However, the combination of content and signal classifiers with the HMM gene structure model is not itself trained to maximize prediction accuracy, and the overall model does not fully account for the statistical dependencies among the features used by the various classifiers. Moreover, recent work on machine learning for structured prediction problems [16,17] suggests that global optimization of model parameters to minimize a suitable training criterion can achieve better results than separate training of the various components of a structured predictor.

To overcome the shortcomings outlined above, our gene predictor uses a linear structure model based on conditional random fields (CRFs) [17], hence the name CRAIG (CRF-based ab initio genefinder). CRFs are discriminatively trained Markovian state models that learn how to combine many diverse, statistically correlated features of the input to achieve high accuracy in sequence tagging and segmentation problems. Our models are semi-Markov [18] to model more accurately the length distributions of genomic regions. For training, instead of the original conditional maximum-likelihood training objective of CRFs, we use the online large-margin MIRA (Margin Infused Relaxed Algorithm) method [19], allowing us to extend to gene prediction the advantages of large-margin learning methods such as support vector machines (SVMs) while efficiently handling very long training sequences. Figure 1 presents schematically the differences in the learning process between our method and the most common generative approach for gene prediction.

**Figure 1:**
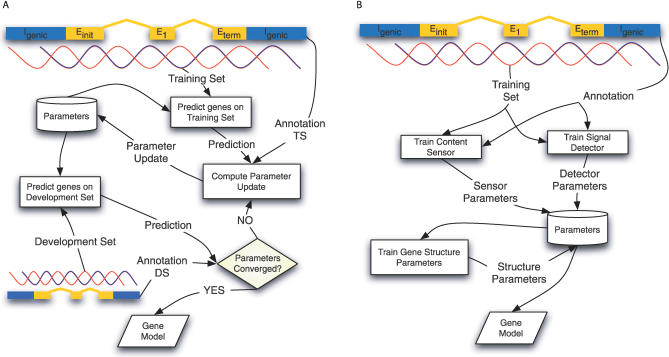
Learning Methods: Discriminative versus Generative Schematic comparison of discriminative (A) and generative (B) learning methods. In the discriminative case, all model parameters were estimated simultaneously to predict a segmentation as similar as possible to the annotation. In contrast, for generative HMM models, signal features and state features were assumed to be independent and trained separately.

Our model and training method allow us to combine a rich variety of possibly overlapping genomic features and to find a global tradeoff among feature contributions that maximizes annotation accuracy. In particular, we model different types of introns according to their length, which would have been difficult to integrate in previous models. We were also able to include rich features for start and stop signals and globally balance their weights against the weights of all other model features. These advances led to significant overall improvements over the current best predictions for the most used benchmark test sets: sensitivity and specificity of initial and single exon predictions showed a relative mean increase [2] of 25.5% and 19.6%, respectively; at the gene level, the relative mean improvement was 33.9%; the relative F-score improvement on the ENCODE regions was 16.05% at the exon level. These improvements were in good part due to the different treatment of intronic states within the model, which in turn increased structure prediction accuracy, particularly on genes with long introns.

Some previous gene predictors have used discriminative training to some extent. HMMGene uses a nongeneralized HMM model for gene structure, which does not include features associated with biological signals, but it is trained with the discriminative conditional maximum likelihood criterion [20]. However, conditional maximum likelihood is more difficult to optimize than our training criterion because it is required to respect conditional independence and normalization for the underlying HMM. GRAPE takes a hybrid approach for learning. It first trains parameters of a generalized HMM (GHMM) to maximize generative likelihood, and then it selects a small set of parameters that are trained to maximize the percentage of correctly predicted nucleotides, exons, and whole genes used as surrogates of the conditional likelihood. This approach is commonly used when training data is limited, and it usually provides superior results only in those cases [21]. However, the GRAPE learning method does not globally optimize the training criterion.

## Results

### Datasets

All the experiments reported in this paper use a gene model trained on a nonredundant set of 3,038 single-gene sequences. We built this set by combining the Augustus training set [2], the GenScan training set, and 1,500 high-confidence CDSs from EnsMart Plus [22], which are part of the Genezilla training set (http://www.tigr.org/software/traindata.shtml). We then appended simulated intergenic material to both ends of each training sequence to make up for the lack of realistic intergenic regions in the training material, as described in more detail in Methods.

We compared CRAIG with GenScan, TwinScan 2.03 (without homology features, also known as GenScan++), Genezilla (formerly known as TigrScan), and Augustus on several benchmark test sets. We also ran predictions with HMMGene, the only other publicly available genefinder to use a discriminative structure training method; we present some prediction results with it in Methods. All programs we compare with are based on similar GHMM models with similar sequence features. Augustus uses two types of length distributions for introns: short intron lengths are modeled with an explicit distribution, but other introns use the default geometric distribution. This difference made Augustus run many times slower than the other programs in all our experiments.

We evaluated the programs on the following benchmark test sets.

#### BGHM953.

This test set combines most of the available single-gene test sets in one single set. It includes the GeneParser I (27 genes) and II (34 genes) datasets [23], 570 vertebrate sequences from Burset and Guigo [24], 178 human sequences from Guigo et al. [25], and 195 human, rat, and mouse sequences from Rogic et al. [26]. Repeated entries were removed. We combined different sets to obtain more reliable evaluation statistics by smoothing out possible overfitting to particular sequence types.

#### TIGR251.

This test set consists of 251 single-gene sequences, which are part of the TIGR human test dataset (http://www.tigr.org/software/traindata.shtml), and it is composed mostly of long-intron genes.

#### ENCODE294.

This test set consists of 31 test regions from the ENCODE project [27,28], for a total of 21M bases, containing 294 carefully annotated alternatively spliced genes and 667 transcripts, after eliminating repeated entries and partial entries with coordinates outside the region's bounds. This is the only test set that was masked using RepeatMasker (http://www.repeatmasker.org/) before performing gene prediction. Table 1 gives summary statistics for the training set and the three test sets.

**Table 1:**
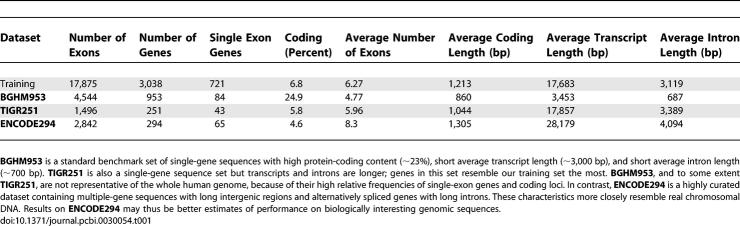
Dataset Statistics

Predictions on all tests and for all programs—including CRAIG—allow partial genes, multiple genes per region, and genes on both strands. Alternative splicing and genes embedded within genes were not evaluated in this work. Any other program parameters were left at their default values. For each program, we used the human/vertebrate gene models provided with the software distributions. In all tests, sequences with noncanonical splice sites were filtered out. Accuracy numbers were computed with the eval package [29], a standard and reliable way to compare different gene predictions.

### Prediction in Single-Gene Sequences

Table 2 shows prediction results for all programs on **BGHM957**. CRAIG achieved better sensitivity and specificity than the other programs at all levels, except for somewhat lower base sensitivity but much higher base specificity than GenScan. The relative F-score improvement for initial and single exons over Genezilla, the second-best program overall for this set, was 14.6% and 5.8%, respectively. Single-exon genes were more difficult to predict for all programs, with specificity barely exceeding 50% for the best program, but CRAIG's relative improvement in sensitivity was nearly 25% over runner-up Genezilla. Terminal exon predictions were also improved over the nearest competitors, but less markedly so. The improved gene-level accuracy follows from these gains at the exon level. GenScan++ and Augustus predicted internal exons with similar accuracy and their F-scores were only slightly worse than CRAIG, but the overall gene-level accuracy for GenScan++ looks much worse because it missed many terminal and single exons. GenScan also did well in this set, but overall performance was somewhat worse than the other programs.

**Table 2:**
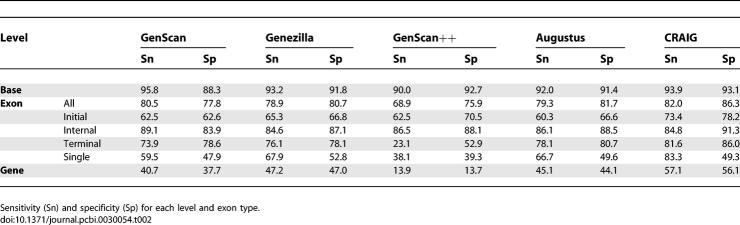
Accuracy Results for **BGHM953**

Most of the genes in this set have short introns and the intergenic regions are truncated, so prediction was relatively easy and all programs did relatively well. The next section compares performance on datasets with long-intron genes and very long intergenic regions.

### Prediction in Long DNA Sequences

As previously noted, **TIGR251** has many genes with very long introns, so it is expected to be harder to predict accurately. This was confirmed by the results in Table 3. Performance was worse for all programs and levels when compared with the first set. However, CRAIG consistently outperformed the other programs with an even wider performance gap than in the first experiment. Here, base and internal-exon accuracies were also substantially improved. CRAIG's relative F-score improvement for bases and internal exons over Genezilla, the second-best program in both categories, was 5.4% and 7.1%, respectively, compared with approximately 1% for **BGHM953**. Other types of exons also improved, as in the first experiment. Because of these better base and exon-level predictions, the relative F-score improvement over runner-up Genezilla at the gene level was about 57%.

**Table 3:**
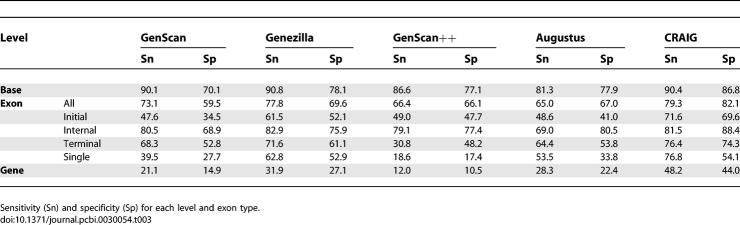
Accuracy Results for **TIGR251**

Our final set of experiments was on **ENCODE294**. The results are shown in Table 4. As previously mentioned, all sequences in this set were masked for low-complexity regions and repeated elements. Unlike previous sets, in which masking did not affect results significantly, prediction on unmasked sequences in this set was worse for all programs (unpublished data). In particular, exon and base specificity decreased an average of 8%.

**Table 4:**
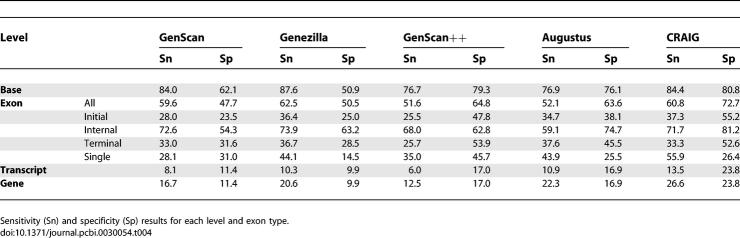
Accuracy Results for **ENCODE294**

We added a transcript-level prediction category to Table 4 to better evaluate predictions on alternatively spliced genes. We closely followed the evaluation guidelines and definitions by Guigo and Reese [28]. There, transcript and gene-level predictions that are consistent with annotated incomplete transcripts are counted correct, even in cases where the predictions include additional exons. We relaxed this policy to also mark as correct those predictions that contained incomplete transcripts whose first (last) exon did not begin (end) with an acceptor (donor). The reason for this change is that no program can exactly predict both ends of such transcripts. We developed our own programs to evaluate single-exon, transcript, and gene-level predictions for incomplete transcripts. Evaluations for other categories and for complete transcripts were handled directly with eval.

To ensure consistency in the evaluation, we obtained all of the programs except for Genezilla from their authors and we ran them on the test set in our lab. Genezilla predictions for this set were obtained directly from the supplementary material provided by [28] so that we could measure the potential differences between our evaluation method and that reported in [28], particularly at the transcript and gene level, for which we expected different results.

Overall, our results for all programs agree with those of Guigo and Reese [28]. Genezilla's base and exon-level results using our evaluation program closely matched the published values. Transcript and gene-level results computed by our method were 1% better than the published numbers, which roughly match the percentage of incomplete annotated transcripts with no splice signals on either end. Computed predictions for GenScan and Augustus were also somewhat different, but not substantially so, from those reported by Guigo and Reese [28], presumably because of differences in program version and operating parameters.

Improvements in this set were similar to those obtained in our second experiment. The relative F-score improvements for individual bases and internal exons were 6% and 15.4% over GenScan++ and Augustus, the runner-ups in each respective category. Improvement in prediction accuracy on single, initial, and terminal exons is similar to that for the other test sets. Transcript and gene-level accuracies were, respectively, 30% and 30.6% better than Augustus, the second-best program overall. This means that our better accuracy results obtained in the first two single-gene sequence sets scale well to chromosomal regions with multiple, alternatively spliced genes.

### Significance Testing

In all tests and at all levels, CRAIG achieved greater improvements in specificity than in sensitivity. We investigated whether the improvements in exon sensitivity achieved by CRAIG could be explained by chance. Any exon belonging to a particular test set is associated with two dependent Bernoulli random variables for whether it was correctly predicted by CRAIG and by another program. We computed *p*-values with McNemar's test for dependent, paired samples from CRAIG and each of the other programs over the three test sets, as shown in Table 5. The null hypothesis was that CRAIG's advantage in exon predictions is due to chance. The *p*-values were <0.05 for all entries, except for the **TIGR251** experiments against Genezilla and the **ENCODE294** experiments against Genezilla and GenScan; in general, these two genefinders proved to be very sensitive at the cost of predicting many more false positives. *p*-Values for the combined test sets were all below 0.001, showing that CRAIG's advantage was extremely unlikely to be a chance event.

**Table 5:**
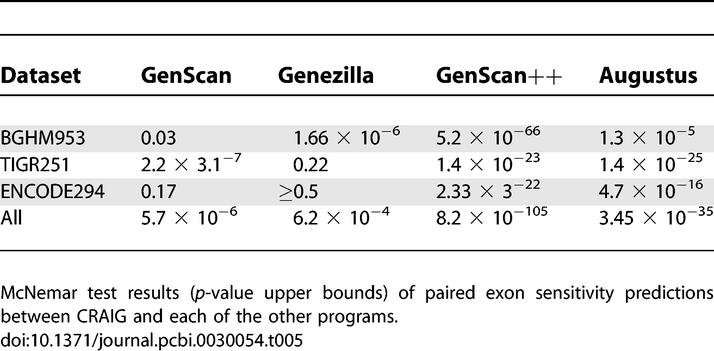
Significance Testing

We also trained and tested an additional variant of CRAIG, in which we did not distinguish between short and long introns; this configuration corresponds closely to the state model representation used in most previous works. Following Stanke and Waack [2], we used the relative mean improvement:

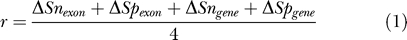
as the measure of differences in prediction accuracy between the CRAIG variant and CRAIG itself. The term Δ*Sn_exon_* denotes the mean increase in exon sensitivity and is defined as

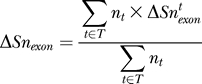
where *n_t_* is the number of annotated genes in dataset *t, T* = {**BGHM953, TIGR251, ENCODE294**}, and Δ*Sn^t^_exon_* is the difference in exon sensitivity between CRAIG and the CRAIG variant on dataset *t*. The other terms are defined similarly. The improvement obtained by CRAIG with respect to the variant was *r* = 3.6. This result was as expected: there was an improvement in accuracy from including the extra intron state in the gene model, but even the simpler variant was more than competitive with the best current genefinders.

## Discussion

It is well-known that more gene prediction errors occur on regions with low GC content, which have higher intron and intergenic region density [15]. This behavior can also be observed on our combined results, as shown in Figure 2A. It also can be noticed that CRAIG had the best F-score for all intron lengths. Except for CRAIG and HMMGene, the F-scores for all other predictors were very close for all lengths. CRAIG's advantage over its nearest competitors became more apparent as introns increased in length. However, all genefinders experience a significant drop in accuracy, at least 25% between 1,000 bp and 16,000 bp. For introns shorter than 1,000 bp, Augustus performs almost as well as CRAIG, in part because of its more complex, time-consuming model for short intron lengths.

**Figure 2:**
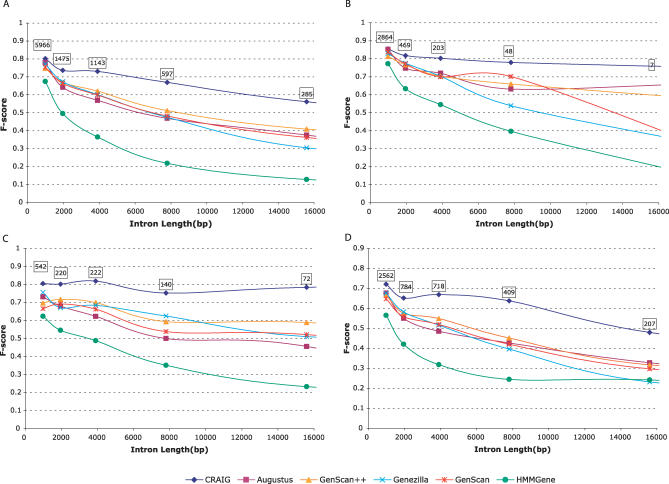
F-Score as a Function of Intron Length Results for all sets combined (A) and for individual test sets shown in subfigures (B–D). The boxed number appearing directly above each marker represents the total number of introns associated with the marker's length. For example, there were 1,475 introns with lengths between 1,000 and 2,000 base pairs for all sets combined (A).

Intron analysis of individual test sets, as shown in Figure 2B–2D, reveals that, except for **ENCODE294**, CRAIG consistently achieved an intron F-score above 75%, even for lengths more than 30,000 bp; in contrast, the F-scores of all other programs fell to lower than 65%, even for introns as short as 8,000 bp. The results show that CRAIG predicts genes with long introns much better than the other programs. This hypothesis was also confirmed with experiments on an edited version of **ENCODE294** in which the original 31 regions were split into 271 contig sequences and all of the intergenic material was deleted except for 2,000 bp on both sides of each gene. This edited version was further subdivided into subsets with—**ALT_ENCODE155**—and without—**NOALT_ENCODE139**—alternative splicing. Figure 3 shows intron prediction results for this arrangement. It can be observed that intron prediction on **NOALT_ENCODE139**, a subset of 139 genes, has the same characteristics as either **TIGR251** or **BGHM953**, that is, a rather flat F-score curve as intron length increases. The same cannot be said about complementary subset **ALT_ENCODE155**, whose significant drop in accuracy for long introns can be explained by the presence of alternative splicing.

**Figure 3:**
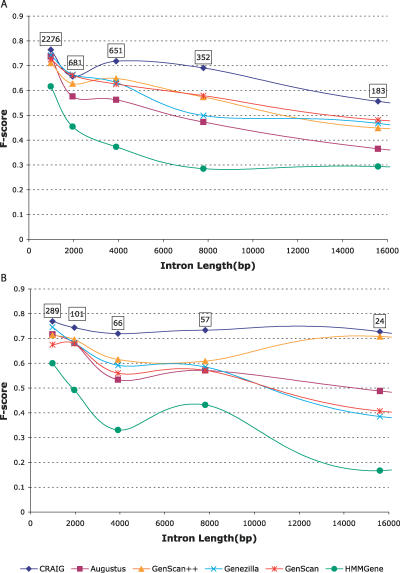
F-Score versus Intron Length for the Encode Test Set Results in subfigures (A) and (B) correspond to the subset of alternatively spliced genes and its complementary subset, respectively.

We claimed in the Introduction that a key aspect of our model and training method is the ability to combine various genomic features and to find a global tradeoff among their contributions so that accuracy is maximized. Being able to identify introns longer than 30,000 bp with prediction accuracy comparable to that achieved on smaller introns is evidence that our program does a better job of combining features to recognize structure. Another way to see how well features have been integrated into the structure model is to examine signal predictions. It is well-known that translation initiation sites (TIS) are surrounded by relatively poorly conserved sequences and are harder to predict than the highly conserved splice signals. Also, stop signals present almost no sequence conservation at all and their prediction depends solely upon how well the last acceptor (in multi-exon genes) or the TIS (in single-exon genes) was predicted. Therefore, a simple splice site classifier can perform fairly well using only local sequence information. In contrast, TIS and stop signal classifiers are known to be much less accurate. Given these observations, we expected CRAIG to improve the most on TIS signal prediction accuracy, as all other programs examined in this work use individual classifiers for signal prediction, whereas CRAIG uses global training to compute each signal's net contribution to the gene structure. Figure 4 shows the improvement in signal prediction accuracy for CRAIG when compared with the second-best program in each case. CRAIG shows improvement for all types of signals, but the improvement was most marked for TIS, especially in specificity. It can also be observed that the improvement on stop signals follows from the co-occurring improvement on both acceptor and TIS signals. The final outcome is that CRAIG makes fewer mistakes in deciding where to start translation and stop translation, which is one of the main reasons for its significant improvement at the gene level.

**Figure 4:**
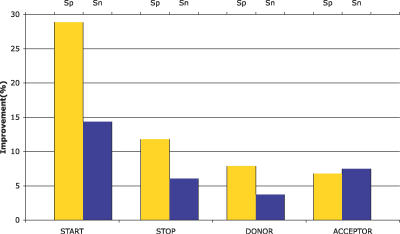
Signal Accuracy Improvements CRAIG's relative improvements in prediction specificity (orange bar) and sensitivity (blue bar) by signal type. In each case, the second-best program was used for the comparison: Genezilla for starts, Augustus for stops, and GenScan++ for splice sites.

There is great potential for including additional informative features into the model without algorithm changes, for instance, features derived from comparative genomics. To facilitate such extensions, we designed CRAIG to allow model changes without recompiling the C++ training and test code. The finite-state model, the features, and their relationships to states and transitions are all specified in a configuration file that can be changed without recompiling the program. This flexibility could be useful for learning gene models on organisms that may require a different finite-state model or a different set of features.

## Materials and Methods

### Gene structures.

In what follows, a gene structure consists of either a single exon or a succession of alternating exons and introns, trimmed from both ends at the TIS and stop signals. We distinguish two different types of introns: short—980 bp or less—and long—more than 980 bp. Figure 5 shows a gene finite-state model that implements these distinctions.

**Figure 5:**
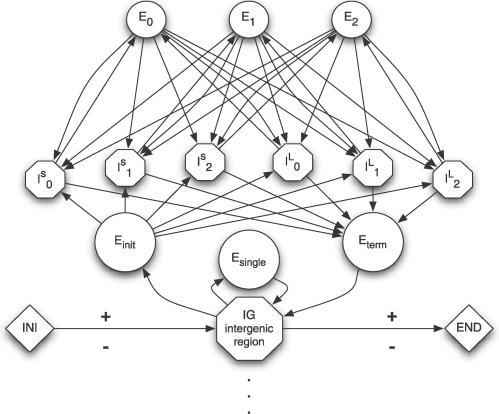
Finite-State Model for Eukaryotic Genes Variable-length genomic regions are represented by states, and biological signals are represented by transitions between states. Short and long introns are denoted by I^S^ and I^L^, respectively.

### Linear structure models.

In what follows, ***x*** *= x*_1_…*x_P_* is a sequence and ***s*** *= s_1_…s_Q_* is a segmentation of ***x***, where each segment *s_j_ =* 〈*p_j_,l_j_,y_j_*〉 starts at position pos(*s_j_*) = *p_j_*, has length len(*s_j_*) = *l_j_*, and state label lab(*s_j_*) = *y_j_*, with *p_j+1_* = *p_j_* + *l_j_* ≤ *P* and 1 ≤ *l_j_* ≤ *B* for some empirically determined upper bound *B*. The training data. 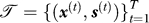 consists of pairs of a sequence and its preferred segmentation. For DNA sequences, *x_i_* ∈ Σ_DNA_ = {A, T, G, C}, and each label lab(*s_j_*) is one of the states of the model (Figure 5). A segment is also referred to as a genomic region; that is, an exon, an intron, or an intergenic region.

A first-order Markovian linear structure model computes the score of a candidate segmentation ***s*** *= s_1_…s_Q_* of a given input sequence ***x*** as a linear combination of terms for individual features of a candidate segment, the label of its predecessor, and the input sequence. More precisely, each proposed segment *s_j_* is represented by a feature vector ***f***(*s_j_,*lab(*s_j−1_*),***x*** *)* ∈ ℜ*^D^* computed from the segment, the label of the previous segment, and the input sequence around position pos(*s_j_*). A weight vector*_,_* ***w*** ∈ ℜ*^D^*, to be learned, represents the relative weights of the features. Then, the score of candidate segmentation ***s*** for sequence ***x*** is given by

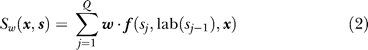



For gene prediction, we need to answer three basic questions. First, given a sequence, ***x*** *,* we need to efficiently find its best-scoring segmentation. Second, given a training set 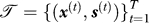 , we need to learn weights ***w*** such that the best-scoring segmentation of ***x*** ^(*t*)^ is close to ***s*** ^(*t*)^. Finally, we need to select a feature function ***f*** that is suitable for answering the first two questions while providing good generalization to unseen test sequences. The next three subsections answer these questions.

### Inference for gene prediction.

Let **GEN(*x*)** be the set of all possible segmentations of ***x***. The best segmentation of ***x*** for weight vector ***w*** is given by:

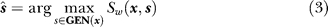



We can compute **ŝ** efficiently from ***x*** using the following Viterbi-like recurrence:

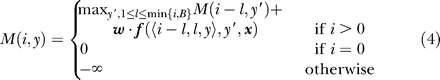



It is easy to see that 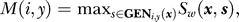 where GEN*_i,y_*(***x***) is the set of all segmentations of *x_1_…x_i_* that end with label *y*. Therefore, *S**_w_***(***x***,***ŝ***) *= M*(*P* + 1,END), where END is a special synchronization state inserted at position *P* + 1. The actual segmentations are easily obtained by keeping back-pointers from each state-position pair (*y,i*) to its optimal predecessor (*y′,i−l*). The complexity of this algorithm is *O*(*PBm* ^2^), where *m* is the number of distinct states and *B* is the upper bound on the segment length, because the runtime of **w** · ***f*** is independent of *P, B,* or *m*. To reduce the constant factor from these dot product computations, most **w** ·***f*** values are precomputed and cached. For introns and intergenic regions, the feature function ***f*** is a sum of per-nucleotide contributions, so the dynamic program in Equation 4 needs only to look at position *i* − 1 when *y* corresponds to such regions. Therefore, *B* needs to be only the upper bound for exon lengths, which was chosen following Stanke and Waack [2]. For long sequences, the complexity of the inference algorithm is therefore dominated by the sequence length *P*.

### Online large-margin training.

Online learning is a simple, scalable, and flexible framework for training linear structured models. Online algorithms process one training example at a time, updating the model weights to improve the model's accuracy on that example. Large-margin classifiers, such as the well-known SVMs, provide strong theoretical classification error bounds that hold well in practice for many learning tasks. MIRA [30] is an online method for training large-margin classifiers that is easily extended to structured problems [19]. Algorithm 1 shows the pseudocode for the MIRA-based training algorithm we used for our models. For each training sequence, ***x*** ^(*t*)^, the algorithm seeks to establish a margin between the score of the correct segmentation and the score of the best segmentation according to the current weight vector that is proportional to the mismatch between the candidate segmentation and the correct one. MIRA keeps the norm of the change in weight vector as small as possible while giving the current example (***x*** ^(*t*)^,***s*** ^(*t*)^) a score that exceeds that of the best-scoring incorrect segmentation by a margin given by the mismatch between the correct segmentation and the incorrect one. The quadratic program in line 5 of Algorithm 1 formalizes that objective, and has a straightforward closed-form solution for this version of the algorithm. Line 11 of the algorithm computes ***w*** as an average of the weight vectors obtained at each iteration, which has been shown to reduce weight overfitting [31]. The training parameter *N* is determined empirically using an auxiliary development set.

**Algorithm 1.** Online Training Algorithm.

Training data 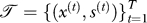 . *L*(***s*** *^(t^* ^)^,***ŝ***) is some nonnegative real-valued function that measures the mismatch between segmentation ***ŝ*** and the correct segmentation ***s*** *^(t^* ^)^ ***.*** The number of rounds *N* is determined using a small development set.

1: ***w*** ^(0)^ = **0**; ***v*** = **0**; *i =* 0
2: **for** round = 1 to *N* **do**
3: **for** *t* = 1 to *T* **do**
4: 
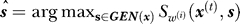

5: 
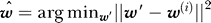

subject to *S**_w′_***(***x*** ^(*t*)^ *,* ***s*** ^(*t*)^) − *S**_w′_***(***x*** ^(*t*)^ *,* ***ŝ***) ≥ *L*(***s*** ^(*t*)^ *,* ***ŝ***)
6: ***w*** ^(*i* + 1)^ ← ***ŵ***
7: ***v*** ← ***v*** + ***w*** ^(*i* + 1)^
8: *i* ← *i + 1*
9: **end for**
10: **end for**
11: ***w*** = ***v*** / (N*T)

Successful discriminative learning depends on having training data with statistics similar to the intended test data. However, this is not the case for gene training data. The main distribution mismatch is that reliable gene annotations available for training are for the most part for single-gene sequences with very small flanking intergenic regions.

To address this problem, we created long training sequences composed of actual genes separated by synthetic intergenic regions as follows. For each training sequence, we generated two extra intergenic regions and appended them to both sequence ends, making sure that the total length of both flanking intergenic regions followed geometric distributions with means 5,000, 10,000, 60,000, and 150,000 bp for each of four GC content classes, respectively [3,10]. The synthetic intergenic regions were generated by sampling from GC-dependent, fourth-order interpolated Markov models (IMMs), with the same form as the models we used to score the intergenic state.

Algorithm 1 also requires a loss function, *L,* and a small development set on which to estimate the number of rounds, *N*. As loss function, we used the correlation coefficient at the base level [24], since it combines specificity and sensitivity into a single measure. The development set consisted of the 65 genes previously used in GenScan [1] to cross-validate splice signal detectors.

### Features.

The final ingredient of the CRAIG model is the feature function ***f*** used to score candidate segments based on properties of the input sequence. A typical feature relates a proposed segment to some property of the input around that segment, and possibly to the label of the previous segment.

*Properties.* We started by introducing basic sequence properties that features are based on. These properties are real-valued functions of the input sequence around a particular position. Some properties represent tests, taking the binary values 1 for true and 0 for false. For any test *P*,‖*P*‖ denotes the function with value 1 if the test is true, 0 otherwise.

The tests

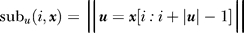
check whether substring ***u*** occurs at position *i* ∈ ***x***. For example, ***x*** = ATGGCGGA would have sub_A_(1,***x***) = 1, sub_TA_(2,***x***) = 0, and sub_GGC_(3,***x***) = 1.

The property score*_y_*(*i,* ***x***) computes the score of a content model for state *y* at position *i*. This score is the probability that nucleotide *i* has label *y* according to a *k*-order interpolated Markov model [32], where *k* = 8 for coding states and *k* = 4 for noncoding states.

The property gcc(*i,* ***x***) calculates the GC composition for the region containing position *i*, averaged over a 10,000-bp window around position *i*.

Each feature associates a property to a particular model state or state transition.

*Binning.* Properties with multimodal or sparse distributions, such as segment length, cannot be used directly in a linear model, because their predictive effect is typically a nonlinear function of their value. To address this problem, we binned each property by splitting its range into disjoint intervals or bins, and converting the property into a set of tests that checked whether the value of the property belonged to the corresponding interval. The effect of this transformation was to pass the property through a stepwise constant nonlinearity, each step corresponding to a bin, where the height of each step was learned as the weight of a binary feature associated to the appropriate test.

For example, following GenScan [1], we mapped the GC content property gcc to four bins: <43, 43–51, 51–57, and >57. For other properties, we used regular bins with a property-specific bin width. For instance, exon length was mapped to 90 bp–wide bins.

*Test and feature combinations.* We used Boolean combinations of tests and binary features to model complex dependencies on the input. Conjunctions can model nucleotide correlations, for example donors of the form G_−1_G_5_, that is, donors with G at positions −1 and 5. Likewise, disjunctions were used to model consensus sequences, for example, donors of the form U_3_, that is, donors with either an A or a G at position 3.

In general, for two binary functions *f* and *g*, we denoted their conjunction by *f* ∧ *g* and their disjunction by *f* ∨ *g*.

### State features.

State features encode the content properties of the genomic regions associated to states: exons, introns, and intergenic regions. State features do not depend on the previous state, so we omitted the previous state argument in these feature definitions.

*Coding/noncoding potential.* This feature corresponds to the log of the probability assigned to the region by the content scoring model:

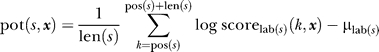
where *μ_y_* is the arithmetic mean of the distribution of log score*_y_* on the training data. For coding regions, the sum is computed over codon scores instead of base scores. Other features related to log score*_y_* also included in ***f*** are the coding differential and the score log-ratios between intronic and intergenic regions.

*Phase biases.* Biases in intron and exon phase distributions have been found and analyzed by Fedorov et al. [33]. We represented possible biases with the straightforward functions

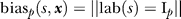
where *p* = 0,1,2 is a phase and I*_p_* is the corresponding intronic state.

*Length distributions.* The length distributions of exons and introns have been extensively studied. Raw exon lengths were binned to allow our linear model to learn the length histogram from the training data. For long introns, with length >980, we used 980/len(*s*) as the length feature, whereas shorter introns used max {245/len(*s*),1}.

For each genomic region type, we also provided length-dependent default features whose weights expressed a bias for or against regions of that length and type. The value of these features is len(s)/*λ_y_*, where *λ_y_* is the average length of all *y*-labeled segments. For introns and intergenic regions, we used separate, always-on default features for the four classes of GC content discussed above.

*Coding composition.* In addition to coding potential scores, which give broad, smoothed statistics for different genomic region types, we also defined count features for each 3-gram (codon) and 6-gram (bicodon) in an exon, and similar count features for the first 15 bases (five codons) of an initial exon. The 3-gram features were further split by GC content class. The general form of such a feature is

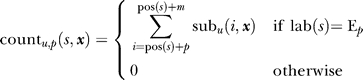
where *p* = 0,1,2 is the phase, *u* is the n-gram, and *m* is the window size, which is len(*s*) for a general exon count, and min{len(*s*),15} for special initial exon features, which attempt to capture composition regularities right after the TIS.

*Masking.* We represented the presence of tandem repeats and other low complexity regions in exonic segments by the function:

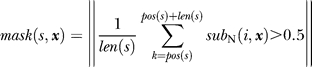
After training, this feature effectively penalizes any exon whose fraction of *N* occurrences exceeds 50% of its total length.

Table 6 shows all the state features associated with each segment label.

**Table 6:**
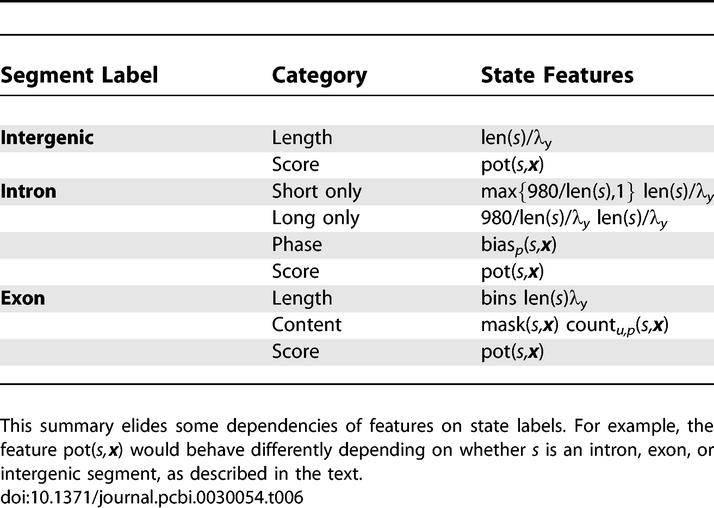
State Features for Each Segment Label

### Transition features.

Transition features look at biological signals that indicate a switch in genomic region type. Features testing for those signals looked for combinations of particular motifs within a window centered at a given offset from the position where the transition occurs. Features of the following form, which test for motif occurrence, are the building blocks for the transition features:

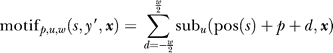
where *p* is the offset, *w* is the window width, and *u* is the motif. This feature counts the number of occurrences of *u* within *p* ± *w*/2 bases of the start of segments.

In principle, all sequence positions are potential signal occurrences, but in practice one might filter out unlikely sites, using a sensitivity threshold proportional to level of signal conservation, thus decreasing decoding time.

Burge and Karlin [1] model positional biases within signals with combinations of position weight matrices (PWMs) and their generalizations, weight array models (WAMs) and windowed weight array models (WWAMs), with very good results. It is straightforward to define these models as sets of features based on our motif*_p,u,w_* feature, as shown here in the WWAM case:

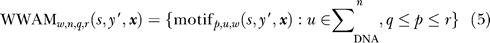



PWMs and WAMs are special cases of WWAMs and can thus be defined by PWM*_q,r_* = WWAM_1,1,*q,r*_ and WAM*_q,r_* = WWAM_1,2,*q,r*_. This means that we can use all of these techniques to model biological signals in CRAIG with the added advantage of having all signal model parameters trained as part of the gene structure.

Correlations between two positions within a signal are captured by conjunctions of motif features. For example, the feature conjunction

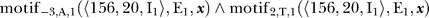
would be 1 whenever there is an A in position −3 and a T in position 2 relative to a donor signal occurring at position 156 in ***x***, and 0 otherwise.

We can also extend the feature conjunction operator to sets of features: if *A* and *B* are sets of features, such as the WWAM defined in Equation 5, we can define the set of features *A* ∧ *B* = {*f* ∧ *g* : *f* ∈ *A, g* ∈ *B*}***.***

In Equation 5, if we use the amino acid alphabet ∑_AA_ instead of ∑_DNA_, and work with codons instead of single nucleotides, we can model signal peptide regions. If we sum over disjunctions of motif features, we can easily model consensus sequences.

Table 7 shows the motif feature sets for each biological signal. The parameters required by each feature type were either taken from the literature [1] or by search on the development set.

**Table 7:**
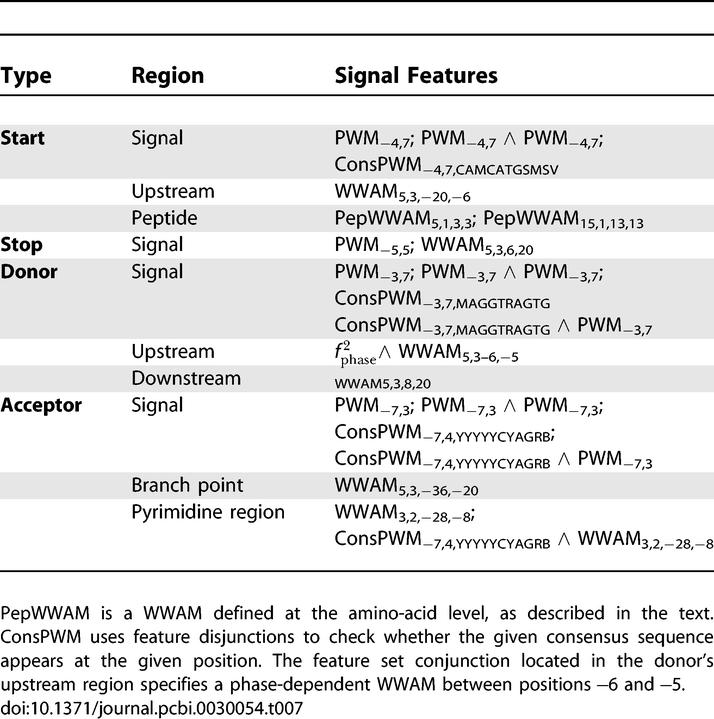
Transition Features per Signal Type

We included an additional feature set, motivated by previous work [2], to learn splice site information from sequences that only contain intron annotations. For any donor (acceptor), we first counted the number of similar donors (acceptors) in a given list of introns. A signal was considered to be similar to another if the Hamming distance between them was at most 1. The features were induced by a logarithmic binning function applied over the total number of similarity counts for Hamming distances 0 and 1.

## Abbreviations

CRAIG: CRF-based ab initio genefinder
CRF: conditional random fields
HMM: hidden Markov model
MIRA: Margin Infused Relaxed Algorithm
PWM: position weight matrices
SVM: support vector machines
TIS: translation initiation site
WAM: weight array model
WWAM: windowed weight array model
